# Reducing antimicrobial resistance through population empowerment: Commentary on: ‘Designing and evaluating a health education session on respiratory infections addressed to caregivers of children under three years of age attending day-care centres in Porto, Portugal: A community-based intervention’

**DOI:** 10.1080/13814788.2016.1276166

**Published:** 2017-03-08

**Authors:** Carl Llor

**Affiliations:** ^a^ University Institute in Primary Care Research Jordi Gol, Primary Healthcare Centre Via RomaBarcelonaSpain

Many steps can be taken to reduce antimicrobial resistance. Since the most important driver for resistance is antibiotic consumption, primary care physicians should reduce unnecessary antibiotic prescription, mainly for respiratory tract infections, for which it continues to be very high in Europe and with vast differences across countries. The majority of resistance-control strategies recommend professional interventions, including point-of-care tests, communication skill training, and interactive patient booklets [1]. Since an association between the number of patients’ visits for infectious episodes and burden of antibiotic prescription has been clearly established, it seems reasonable that limiting the number of provider visits for these conditions would result in a reduction of antibiotic prescribing. For instance, in a 12-year study carried out in the US, Grijalva et al., observed a sustained decrease in antibiotics prescribed for acute respiratory tract infections in children under five years of age, with this reduction being mainly associated with a reduction of visit rates for otitis media [2]. Other observational studies have also found this association between consultation rates for these conditions and antibiotic prescribing [3,4].

This issue of *the European Journal of General Practice* includes an original Portuguese community-based study encompassing different steps aimed at improving the knowledge of acute upper respiratory tract infections among caregivers working in day-centre nurseries with children under three years of age [5]. The authors gathered information about the needs of the caregivers on different topics using a Delphi method, and once these needs had been identified, they carried out a randomized clinical trial evaluating the effectiveness of a health education session covering the aspects raised in this first analysis. The intervention resulted in significantly better outcomes as caregivers assigned to intervention answered questions about knowledge and attitudes about acute respiratory infections more appropriately than those assigned to the control group. In addition, intervened caregivers were able to perform nasal irrigation and use nasal aspirators and nebulization much more appropriately than their counterparts. The effectiveness of steam inhalation and nebulizations is controversial but there is some evidence about the benefit of using saline nasal irrigation for upper respiratory tract infections. In a recent controlled trial, Little et al., observed that advice to use nasal irrigation in routine primary care for adults with chronic or recurrent sinusitis resulted in lower symptom scores, reduced use of medications and, most importantly, less need to consult primary care physicians in future episodes [6].

Since most acute respiratory tract infections are self-limited and can be managed with over-the-counter symptomatic medications, patient empowerment in self-management seems crucial. Also, this empowerment should not only address the self-management of these infections but also correct the misconceptions many people have about the role of antibiotics for illnesses for which these drugs are not helpful, such as the common cold or influenza. Therefore, adequate patient education is required to adjust the widespread belief among patients that antibiotics are a solution for nearly all infections. GPs should also give the public clear information about prevention, recommending hygiene such as handwashing, and the duration of symptoms, with the aid of leaflets for instance. A recent review provided evidence that the use of patient information leaflets on common infections during GP consultation may effectively reduce antibiotic prescriptions and antibiotic use as well as patients’ intentions to re-consult [7]. The use of the delayed prescribing of antibiotics is another example of patient empowerment. This method, whereby a prescription is issued by a health professional for use by the patient at a later date if their symptoms do not improve, has proved to reduce safely the amount of antibiotics consumed by patients. Despite causing minor reductions in patient satisfaction, a systematic review of randomized clinical trials found that only 28% of the patients used antibiotics if they had to return to collect a delayed prescription from the centre reception, without increasing rates of complications or re-consultations [8].

Changing the behaviour and the expectations of the general population along with increasing their knowledge about the management of respiratory tract infections, mainly that related to symptomatic treatment, could have a huge influence on decreasing the number of provider visits. A recent study found that 72% of the primary care visits due to an acute respiratory tract infection did not seem to require an office visit [9]. If we enable patients and caregivers to increase their knowledge about self-management of respiratory tract infections, as in the paper published by Alexandrino et al., fewer visits to GPs and paediatricians will be expected. Fewer visits to healthcare providers means a lower number of antibiotics, and both mean lower healthcare costs and lower resistance rates.
